# Lethal and Sublethal Effects of Conventional and Organic Insecticides on the Parasitoid *Trissolcus japonicus*, a Biological Control Agent for *Halyomorpha halys*

**DOI:** 10.3389/finsc.2021.685755

**Published:** 2021-06-14

**Authors:** Arthur V. Ribeiro, Sarah G. Holle, William D. Hutchison, Robert L. Koch

**Affiliations:** Department of Entomology, University of Minnesota, Saint Paul, MN, United States

**Keywords:** brown marmorated stink bug, exposure routes, fecundity, fertility, longevity, samurai wasp, sex

## Abstract

The egg parasitoid *Trissolcus japonicus* is a natural enemy of *Halyomorpha halys*, a polyphagous invasive pest in Europe and North and South America. Integration of chemical and biological control tactics could facilitate effective and sustainable integrated pest management programs. This study was conducted to assess (i) the lethal effects of field rates, (ii) the sublethal effects of maximum and half field rates, and (iii) the lethal effects of different routes of exposure of three organic and two conventional insecticides against *T. japonicus*. Maximum field rates of spinosad and sulfoxaflor resulted in acute lethal toxicity to adult *T. japonicus* 1 week after residual contact exposure. Maximum and half field rates of pyrethrins, the mixture of azadirachtin and pyrethrins, and clothianidin caused sublethal effects to female wasps through residual contact exposure. Furthermore, all insecticides caused acute lethal effects 1 week after ingestion by unmated female wasps. Taken together, these results suggest that careful planning is necessary to ensure compatibility between biological and chemical control for *H. halys*. The insecticides evaluated in this study varied in toxicity to *T. japonicus* and should be used with caution to conserve this natural enemy for biological control of *H. halys*.

## Introduction

The brown marmorated stink bug, *Halyomorpha halys* (Stål) (Hemiptera: Pentatomidae), became a major concern worldwide in the last two decades as a nuisance and agricultural pest because it aggregates and overwinters in human structures (1), and is a highly polyphagous insect that feeds on more than 200 host plants (2). This insect originated in East Asia and it has invaded Europe, Oceania and North and South America, with the potential for continued spread (3–5). In the United States, *H. halys* is established across much of the country, causing great economic losses to fruit, vegetable and field crops (5–7).

In its region of origin, *H. halys* populations are naturally regulated by organisms such as predators and parasitoids (7, 8). Among these natural enemies, the egg endoparasitoid, *Trissolcus japonicus* (Ashmead) (Hymenoptera: Scelionidae), has been identified as an effective control agent of *H. halys* (9, 10) with parasitism rates up to 70% (8). Adventitious populations of this parasitoid have been reported occurring in eastern and western parts of the United States (9–11), with potential of further expanding its distribution within the country, including the upper Midwest (12, 13). In the United States, the natural biological control of *H. halys* by native species seems to be insufficient to keep populations of this pest under control (7, 14–16). Thus, studies are exploring *T. japonicus* as a candidate for classical and/or augmentative biological control of *H. halys* (7, 10, 13).

As with many invasive species, chemical control is still largely relied upon as the main control tactic to suppress pests (17–19), including *H. halys* (7) in agroecosystems. However, the excessive use of insecticides, notably broad-spectrum insecticides, can endanger the environment and non-target organisms, and lead to the development of resistant insect populations (20, 21). In the context of integrated pest management (IPM), the integration of different control tactics (e.g., biological and chemical controls) is preferable to mitigate the negative impacts of pest control and achieve more sustainable crop production (21). Furthermore, the success of biological control programs often depends on the compatibility of the natural enemies with other management practices including chemical control (18, 22).

Increasing concern with the adverse effects of insecticides has led to the development of more selective synthetic insecticides (17) and expansion of environmentally safer production systems such as organic farming where the use of synthetic products is restricted (23). However, compounds registered to organic production with lethal and sublethal effects to beneficial insects have also been documented in the literature (24, 25). For instance, organic insecticides have been characterized as causing mortality, reducing hatchability, and longevity of parasitoids of stinkbug eggs (26).

Insecticides can harm insects through different routes of exposure (e.g., residual contact and/or oral exposure) (18, 27). In addition, insects may be exposed to different concentrations of insecticides under field conditions due to non-uniform application and degradation of the products after application (17, 25). These variable levels of exposure can result in natural enemies experiencing different lethal and sublethal effects (18, 25). Therefore, these aspects must also be assessed for a more complete understanding of the potential impacts of insecticides on non-target organisms, such as natural enemies (25).

To advance biological control of *H. halys*, the impacts on *T. japonicus* of insecticides used for the management of *H. halys* must also be investigated. The lethal and sublethal effects of some synthetic and organic insecticides on *T. japonicus* have been recently investigated (28). However, there is still a lack of information regarding the effects of different concentrations and routes of exposure to this natural enemy. Therefore, this study was conducted to assess (i) the lethal effects of field rates, (ii) the sublethal effects of maximum and half field rates, and (iii) the lethal effects of different routes of exposure of three organic and two conventional insecticides against *T. japonicus*. This study will help to improve management practices that conserve *T. japonicus* and ensure its efficacy under conventional and organic farming.

## Materials and Methods

### Insect Stock Colony

*Trissolcus japonicus* originating from Beijing, China, were reared in the Biosafety Level 2 (BSL-2) Insect Biocontrol Facility at the University of Minnesota, Saint Paul campus, Minnesota, United States, as described in Nystrom Santacruz et al. (13). Insects were kept in 9-dram polystyrene plastic vials inside incubators (Intellus Environmental Controller, Percival Scientific, Perry, IA, United States) under 23°C, 16:8 h light:dark, and 60–65% relative humidity conditions. Honey was provided for nutrition and adults were allowed to oviposit on *H. halys* egg masses. Egg masses of *H. halys* came from a University of Minnesota laboratory colony [protocol described in Pezzini et al. (15)] and were stored in a −80°C ultralow freezer. Egg masses <2 months old were thawed for at least 15 min before being added to the vials (one egg mass per vial) with mated *T. japonicus* for oviposition (29). Mated *T. japonicus* were allowed to oviposit for 72 h and parasitized egg masses were individualized into new vials. A small drop of honey was added under the lid of each vial containing a parasitized egg, with the help of a wooden dowel (3.2 mm width), on the day prior to the emergence of adults. Then, this standard procedure was repeated once for each *T. japonicus* generation.

### Lethal Effect of Insecticides on *T. japonicus*

This bioassay was conducted as an overall assessment of the residual contact toxicity of insecticides to pooled *T. japonicus* males and females. For the subsequent more detailed experiments, only females were used because of their importance from a population perspective. To provide a broad representation of production systems, three insecticides used in organic farming (spinosad [Entrust®, Corteva Agriscience], pyrethrins [Pyganic®, McLaughlin Gormley King Company], and the mixture of azadirachtin and pyrethrins (azadirachtin + pyrethrins) [Azera®, McLaughlin Gormley King Company)] and two synthetic insecticides (sulfoxaflor [Transform®, Corteva Agriscience] and clothianidin [Belay®, Valent BioSciences)] were selected for use in this experiment. For treatments, insecticides were mixed with distilled water at their respective maximum recommended field rates (Table 1) and distilled water with no insecticide was used as the control. A completely randomized design with 14 replicates was used for this experiment. The experimental unit was a polystyrene plastic (33.7 mL) vial sealed with its original white plastic cap (henceforth referred to as sealed polystyrene plastic vial), and containing a strip of insecticide-treated filter paper (11.87 cm^2^) (Fisherbrand Filter paper P8, cut in half), four *T. japonicus* adults (two males and two females) <48 h old and honey for nutrition. Honey was provided by adding a small drop of honey under the lid of each vial with the help of a wooden dowel (3.2 mm width). Residual contact exposure was conducted using insecticide-treated filter paper with methods adapted from Campbell et al. (30) and Ogburn and Walgenbach (26). Filter paper strips were treated by evenly pipetting 0.04 mL of distilled water-insecticide solution (or distilled water for the control) over the strip of filter paper. The strips of treated filter paper were allowed to dry for 1 h before being placed in the individual vials. In a fume hood, the adults were transferred using an aspirator to the vials containing the insecticide-treated filter paper. Treatment vials were held in the same conditions as the colony. Survival was assessed after 7 days and was defined as parasitoids displaying coordinated movement, such as being able to walk at least one body length.

**Table 1:** Insecticides (product names and active ingredients), production systems where they are adopted, and maximum recommended field rates used in the study.

**Product**	**Active ingredient**	**Production system**	**Field rate**
PyGanic®	Pyrethrins	Organic	4.69 L/ha
Azera®	Azadirachtin + Pyrethrins	Organic	4.09 L/ha
Belay®	Clothianidin	Conventional	0.88 L/ha
Transform®	Sulfoxaflor	Conventional	192.65 g/ha
Entrust®	Spinosad	Organic	0.73 L/ha

### Sublethal Effects on *T. japonicus* Longevity

Sublethal effects on longevity were assessed for unmated *T. japonicus* females with residual contact exposure to pyrethrins, azadirachtin + pyrethrins, and clothianidin (Table 1). Unmated females were used because a reduction in longevity associated with mating has been observed for another parasitoid, *Trichogramma minutum* (Hymenoptera: Trichogrammatidae) (31). This subset of insecticides did not differ statistically from the control in the lethal effects experiment described in section Lethal Effect of Insecticides on *T. japonicus*. For treatments, rates of 100 and 50% of the maximum recommended field rate were tested for each insecticide mixed with distilled water, and distilled water with no insecticide was used as the control. As adults emerged, they were isolated with the help of an aspirator into separate vials with honey for nutrition. A completely randomized design with 30 replicates was used. The experimental unit was a sealed polystyrene plastic vial (33.7 mL) with a strip of treated filter paper, one *T. japonicus* unmated female <48 h old and honey for nutrition. The experimental conditions, treatment of filter papers and transferring of insects were conducted as described in section Lethal Effect of Insecticides on *T. japonicus*. Survival, as defined above, was evaluated three times per week until all individuals died.

### Sublethal Effects on *T. japonicus* Reproduction

Sublethal effects on reproduction were assessed for *T. japonicus* with residual contact exposure to pyrethrins, azadirachtin + pyrethrins, and clothianidin (Table 1). For treatments, rates of 100 and 50% of the maximum recommended field rate were tested for each insecticide mixed with distilled water, and distilled water with no insecticide was used as the control. The experiment was performed over three temporal blocks, with each block consisting of 10 mated females per treatment. Mated females were obtained by transferring newly emerged male and female adults <48 h old, with the help of an aspirator, to separate vials with honey for nutrition. Two males and no more than four females per vial were allowed to mate for 72 h prior to setting up the experiment. Then, mated females were transferred to the vials containing the insecticide-treated strip of filter paper. Thus, the experimental unit was a sealed polystyrene plastic (33.7 mL) vial with a strip of treated filter paper, one *T. japonicus* mated female and honey for nutrition. The experimental conditions, treatment of filter papers and transferring of insects were conducted as described in section Lethal Effect of Insecticides on *T. japonicus*. Survival, as described above, of mated females was recorded at 1, 3, 5, 7, 9, 14, 16, 21, and 23 days.

Thawed *H. halys* egg masses <2 months old were added to the treatment vials at 1, 3, 7, 14, and 21 days and the females were allowed to oviposit for 48 h on each egg mass. All the egg masses had a similar number of eggs (27.78 ± 1.33 [average ± standard deviation] eggs/egg mass), and egg masses were randomly assigned to each female. After this period, egg masses were removed and were placed in individual vials to be monitored for adult emergence. The egg masses were monitored for 6 weeks after exposure to the females. Emerged adults were sexed and counted for each egg mass. Unhatched eggs were dissected under a stereomicroscope (8x−35x magnification, Leica EZ 4, Leica Microsystems, Switzerland) with the help of a needle (#2 insect pin) and egg status was characterized as complete parasitism (fully developed wasp that failed to emerge for unknown reasons), incomplete parasitism (underdeveloped wasp) or unparasitized (eggs that had no evidence of parasitism) (14, 32, 33). Proportion of female emergence was calculated as the proportion of females out of the total emerged adults per egg mass per female. Fertility was calculated as the cumulative number of parasitized eggs (i.e., eggs from which wasps emerged, or with complete or incomplete parasitism as determined through dissection) per female. Fecundity was calculated as the number of offspring produced (i.e., those successfully emerged) per female.

### Lethal Effect of Insecticides Through Different Exposure Routes

This experiment was conducted to determine the lethal effect of the insecticides used in the first experiment (Table 1) to unmated females by residual contact, oral or both residual contact and oral exposure routes. For treatments, 100% of the maximum field rate of each insecticide was mixed with distilled water or with a 10% sucrose solution (w/v), for the residual contact and oral exposure routes, respectively. Distilled water and 10% sucrose solution with no insecticide were used as the control. Treatments consisted of a combination of untreated 10% sucrose solution with a strip of insecticide-treated filter paper (residual contact), insecticide-treated 10% sucrose solution with a strip of untreated filter paper (oral), and insecticide-treated 10% sucrose solution with a strip of insecticide-treated filter paper (both residual contact and oral) for each insecticide. The 10% sucrose solution was provided for nutrition by pipetting 0.04 mL of the solution (insecticide-treated or untreated) on 1-cm diameter cotton ball placed inside a 2-mL Eppendorf microtube to avoid its direct contact with the filter paper. The mictotubes were then added to the polystyrene plastic vial (33.7 mL) containing the filter paper (insecticide-treated or untreated).

Treatment of filter paper was done following the procedures described in section Lethal Effect of Insecticides on *T. japonicus*. Unmated females were obtained as described in section Sublethal Effects on *T. japonicu*s Longevity. The experiment was performed over three temporal blocks, with each block containing 10 unmated females <48 h old per treatment in blocks one and three, and five unmated females <48 h old per treatment in block two. The experimental conditions and transferring of insects were conducted as described in section Lethal Effect of Insecticides on *T. japonicus*. The cotton ball with 10% sucrose solution was removed after 48 h to avoid mold growth on the cotton. For this reason, the strip of filter paper was also removed from all vials after 48 h to avoid a possible confounding effect of different durations of exposure. After removal of the cotton ball, fresh honey was provided as described in section Lethal Effect of Insecticides on *T. japonicus*. Survival, as described above, was evaluated daily for 1 week.

### Statistical Analyses

All analyses and graphs were obtained using the software R version 3.5.1 (34) and RStudio Desktop version 1.1.463 (35). The proportion of surviving adults from the residual contact toxicity of insecticides to pooled *T. japonicus* males and females was submitted to a biased-reduced generalized linear model [R package, *command*: stats, *glm*; (34)] with a binominal distribution (logit link). A mean bias-reduced adjustment was accomplished using the “brglmFit” method (in the glm function), from the “brglm2” package (36), to account for complete separation of the data. Survival was included as the response and treatments as the explanatory variable. The overall effect of each parameter was estimated with a likelihood-ratio chi-square test [car, *Anova*; (37)]. Means were compared using the Tukey's test (*P* < 0.05) [multcomp, *ghlt*; (38)].

Data on survival over time were analyzed using the Kaplan-Meier estimation method to obtain survival curves [survival, *survfit*; (39)]. Survival curves were then compared by the log-rank test (*P* < 0.05) [survival, *survdiff* ; (39)]. This procedure was used to check the effect of blocks (i.e., for each treatment and route of exposure). This effect was overall non-significant in all experiments, so data from different blocks were combined. For the experiment testing different routes of exposure, survival curves were constructed using two approaches: (i) survival of exposure routes by treatment, and (ii) survival of treatments by exposure route.

Proportion of female emergence data were analyzed using a generalized linear mixed model [lme4, *glmer*; (40)] with a binominal distribution (logit link), and time, treatments, blocks and the interaction between time and treatments as fixed factors. Wasp (F0) was included as a random factor to account for repeated measures of individual wasps over time. The significance of each fixed factor was estimated with a Type II Wald chi-square test [car, *Anova*; (37)]. Model fit was verified with diagnostic plots of scaled residuals against fitted values and with goodness-of-fit tests on the scaled residuals [DHARMa; (41)].

Data on fertility (i.e., mean cumulative number of eggs parasitized by the final date of the experiment) and fecundity (i.e., mean cumulative number of successfully emerged offspring by the final date of the experiment) were analyzed with separate unbalanced analyses of variance [car, *Anova*; (37)] using general linear models [stats, *lm*; (42)] with treatments and blocks as the explanatory variables. The interactions between treatments and blocks were not significant and were therefore dropped from the models. Means were compared using Tukey's test at *P* < 0.05 [agricolae, *HSD.test*; (43)]; and the normality of errors and homogeneity of variances were checked using qqplots, and plots of the residuals against fitted values. Three outliers (coming from early deceased wasps) were identified in the diagnostic plots and, for this reason, were removed from the fertility, fecundity and proportion of female emergence data. Models followed assumptions after the removal of the outliers.

## Results

### Lethal Effect of Insecticides on *T. japonicus*

**Figure 1:**
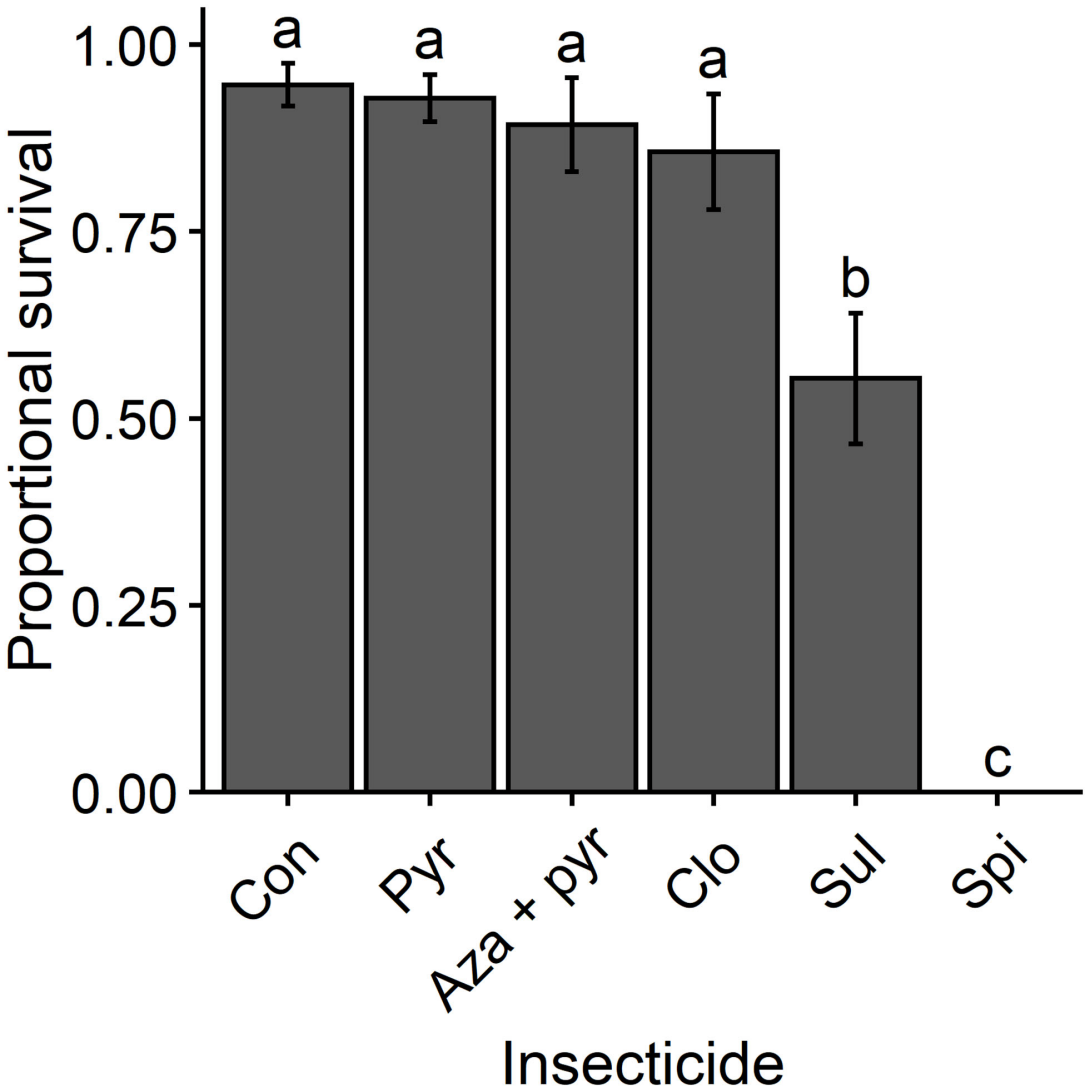
Mean proportional survival (± SEM) of *T. japonicus* 7 days after residual contact exposure to the maximum recommended field rates of five insecticides and an untreated control (i.e., distilled water). Different letters above bars indicate differences among treatments according to the Tukey's test (*P* < 0.05). Con, control; Pyr, pyrethrins; Aza + pyr, azadirachtin + pyrethrins; Clo, clothianidin; Sul, sulfoxaflor; Spi, spinosad.

Proportional survival rates of *T. japonicus* adults differed significantly following residual contact exposure to different treatments (χ^2^ = 198.10, df = 5, *P* < 0.001). Survival was lowest for *T. japonicus* exposed to residues of the field rate of spinosad and intermediate for those exposed to the field rate of sulfoxaflor (Figure 1). Survival of *T. japonicus* exposed to residues of the field rates of pyrethrins, azadirachtin + pyrethrins and clothianidin did not differ from that of the untreated control (Figure 1).

### Sublethal Effects on *T. japonicus* Longevity

**Figure 2:**
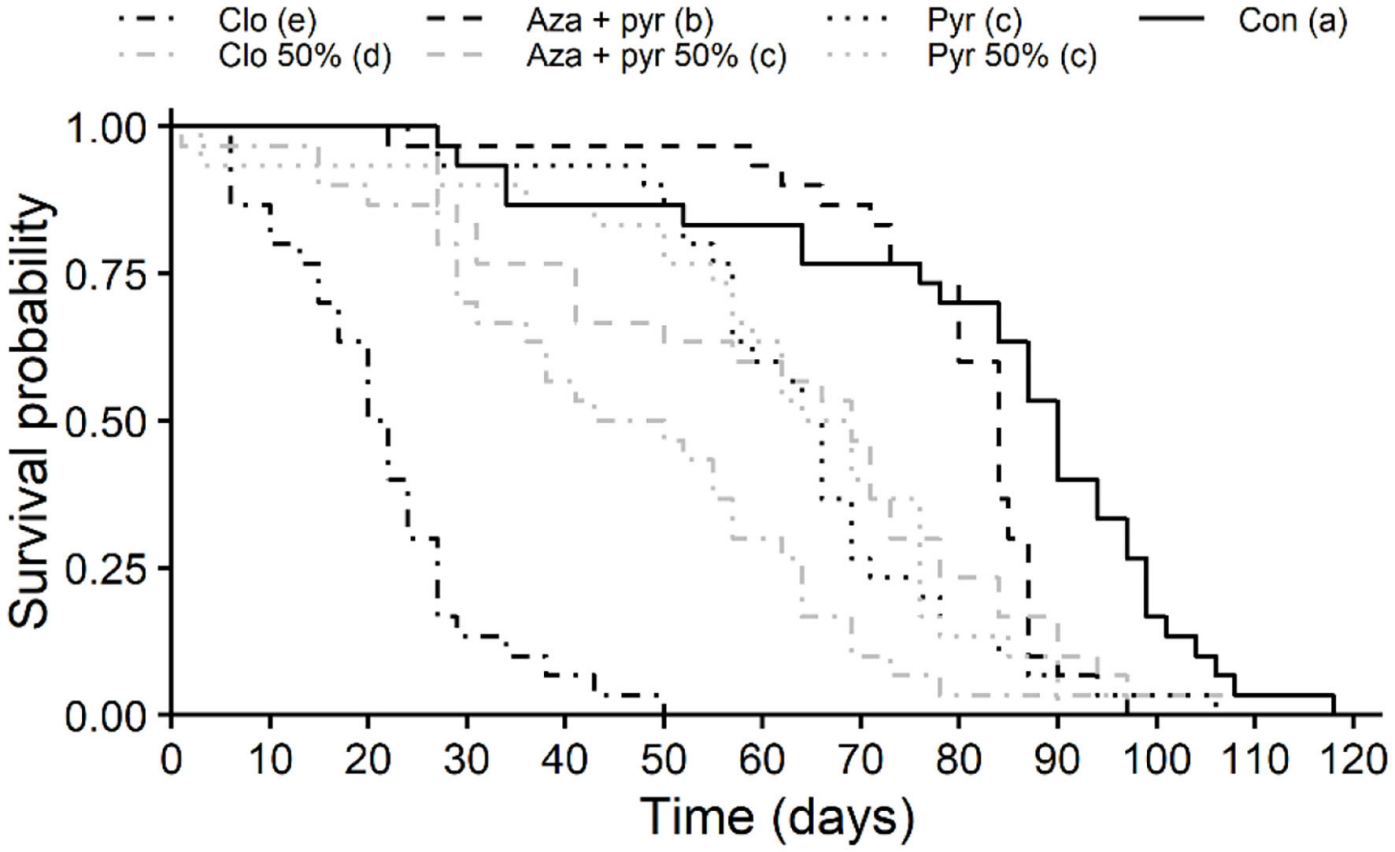
Longevity (Kaplan-Meier survival probability curves) of *T. japonicus* unmated females with residual contact exposure to 100 and 50% of the maximum field rates of three insecticides and an untreated control (distilled water). Different letters in parentheses in the legend indicate differences among survival curves according to the Log-Rank test adjusted with the Benjamini-Hochberg method (*P* < 0.05). Con, control; Pyr, pyrethrins; Aza + pyr, azadirachtin + pyrethrins; Clo, clothianidin.

Survival curves of unmated *T. japonicus* females differed significantly after residual contact exposure to different treatments (χ^2^ = 219, df = 6, *P* < 0.001). Survival was lowest for the field rate of clothianidin followed by the 50% rate of this insecticide (Figure 2), with longevities (i.e., time to 50% mortality [LT_50_]) of 23.33 and 51.66%, respectively, of that of the control (Table 2). Survival of *T. japonicus* exposed to the 50% rate of azadirachtin + pyrethrins and both rates of pyrethrins were intermediate, with longevities (i.e., LT_50_s) of 72.22–76.66% of that of the control. Survival for the field rate of azadirachtin + pyrethrins was greater than the other insecticides, but less than the control (Figure 2), with longevity (i.e., LT_50_) of 93.33% of that of the control (Table 2).

**Table 2:** Lethal time until the death of 50% of *T. japonicus* unmated females (LT_50_) for the control (i.e., distilled water) and selected insecticides.

Treatment^*^	**Tested rate (L/ha)**	***n***	**LT_**50**_ (days)**	**95% Confidence interval**
Con		30	90.00	84.00–97.00
Pyr	4.69	30	65.00	57.00–69.00
	2.35	30	66.50	59.00–76.00
Aza + pyr	4.09	30	84.00	80.00–87.00
	2.05	30	69.00	50.00–78.00
Clo	0.88	30	21.00	17.00–27.00
	0.44	30	46.50	36.00–62.00

* *Con, control; Pyr, pyrethrins; Aza + pyr, azadirachtin + pyrethrins; Clo, clothianidin*.

### Sublethal Effects on *T. japonicus* Reproduction

**Figure 3:**
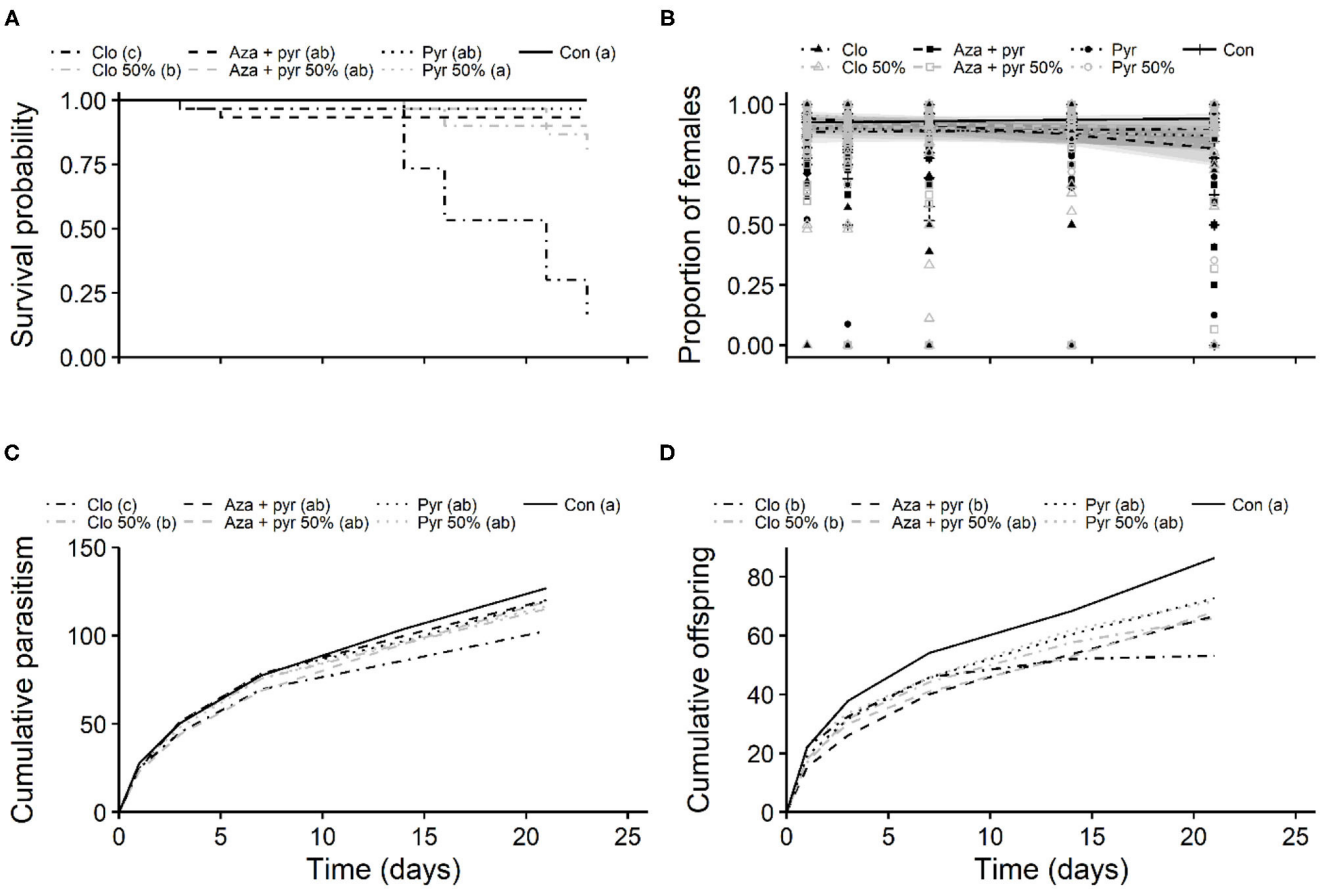
Kaplan-Meier survival probability curves of mated females **(A)**, and proportion of emerged females per egg mass **(B)**, mean cumulative parasitism (fertility) **(C)**, and mean cumulative number of offspring (fecundity) **(D)** per mated female of *T. japonicus* after residual contact exposure to 100 and 50% of the maximum recommended field rates of three insecticides and an untreated control (distilled water). Different letters in parentheses in the legend indicate differences among treatments (*P* < 0.05) according to: **(A)** the Log-Rank test adjusted with the Benjamini-Hochberg method; **(C,D)** the Tukey's test. Note that the survival probability curve of Pyganic 50% is completely overlapped by the control. Con, control; Pyr, pyrethrins; Aza + pyr, azadirachtin + pyrethrins; Clo, clothianidin.

Survival curves of mated *T. japonicus* females differed significantly after residual contact exposure to different treatments (χ^2^ = 141, df = 6, *P* < 0.001). Survival was lowest for the field rate of clothianidin followed by the 50% rate of this insecticide. Survival of *T. japonicus* exposed to the field rate of pyrethrins and both rates of azadirachtin + pyrethrins were greater than that of both rates of clothianidin, but less than the 50% rate of pyrethrins and the control (Figure 3A).

The mean number of emerged and unemerged eggs per egg mass per treatment are described in Table 3. Mean proportion of emerged females per egg mass per female did not differ significantly after residual contact exposure to different treatments (χ^2^ = 11.05, df = 6, *P* = 0.087), but significant effects of blocks (χ^2^ = 15.06, df = 2, *P* < 0.001), time (χ^2^ = 40.92, df = 1, *P* < 0.001), and the interaction between treatments and time (χ^2^ = 58.64, df = 6, *P* < 0.001) were observed (Figure 3B). Overall, the proportion of females per egg mass varied from 0.81 to 0.89 (Figure 3B). The odds of female emergence decreased over time, with a higher rate of decrease observed for the field rate of azadirachtin + pyrethrins and the 50% rate of pyrethrins (Figure 3B).

**Table 3:** Mean number of emerged *T. japonicus* and unemerged eggs per egg mass per mated female (± SEM) over a period of 7 days after residual contact exposure to insecticides at 100 or 50% of their maximum recommended field rates (distilled water was used as the control).

Treatment^*^	**N^†^**	**Emerged**	**Unemerged^‡^**		
			**Complete**	**Incomplete**	**Unparasitized**
Con	150	17.26 ± 0.90	0.69 ± 0.11	7.19 ± 0.80	2.25 ± 0.46
Pyr 100%	148	14.73 ± 0.96	0.77 ± 0.11	8.47 ± 0.82	3.87 ± 0.65
Pyr 50%	149	14.44 ± 0.98	0.72 ± 0.13	8.15 ± 0.86	4.68 ± 0.70
Aza + pyr 100%	141	14.05 ± 1.00	0.96 ± 0.15	9.01 ± 0.89	3.69 ± 0.68
Aza + pyr 50%	146	13.99 ± 0.96	0.91 ± 0.14	8.94 ± 0.90	3.95 ± 0.62
Clo 100%	116	13.74 ± 1.12	0.82 ± 0.17	7.18 ± 0.93	6.15 ± 0.88
Clo 50%	141	13.76 ± 0.99	0.92 ± 0.15	8.25 ± 0.88	4.42 ± 0.68

* *Con, control; Pyr, pyrethrins; Aza + pyr, azadirachtin + pyrethrins; Clo, clothianidin*.

† *Number of H. halys egg masses*.

‡ *Eggs from which wasps did not emerge were classified as containing fully developed wasps that failed to emerge (complete), underdeveloped wasps (incomplete), or eggs that were not parasitized (unparasitized)*.

Mean cumulative fertility of mated *T. japonicus* females differed significantly among insecticides (F_6, 198_= 14.78, *P* < 0.001) and blocks (F_2, 198_= 31.57, *P* < 0.001) after residual contact exposure to different treatments. The fertility of mated *T. japonicus* ranged from 86.93 to 126.87 (average ± standard error: 113.54 ± 1.59) and it was lowest for the field rate of clothianidin, followed by the 50% rate of this insecticide (Figure 3C). Fertility of *T. japonicus* exposed to both rates of pyrethrins and azadirachtin + pyrethrins did not differ from that of the control (Figure 3C). Similarly, both treatments (F_6, 198_ = 4.71, *P* < 0.001) and blocks (F_2, 198_ = 11.19, *P* < 0.001) affected the mean fecundity of mated *T. japonicus* females. The mean cumulative number offspring per female ranged from 54.96 to 86.43 (average ± standard error: 70.04 ± 1.78) and it was lowest for both rates of clothianidin and the field rate of azadirachtin + pyrethrins (Figure 3D). Both rates of pyrethrins and the 50% rate of azadirachtin + pyrethrins did not differ from the control (Figure 3D).

### Lethal Effect of Insecticides Through Different Exposure Routes

**Figure 4:**
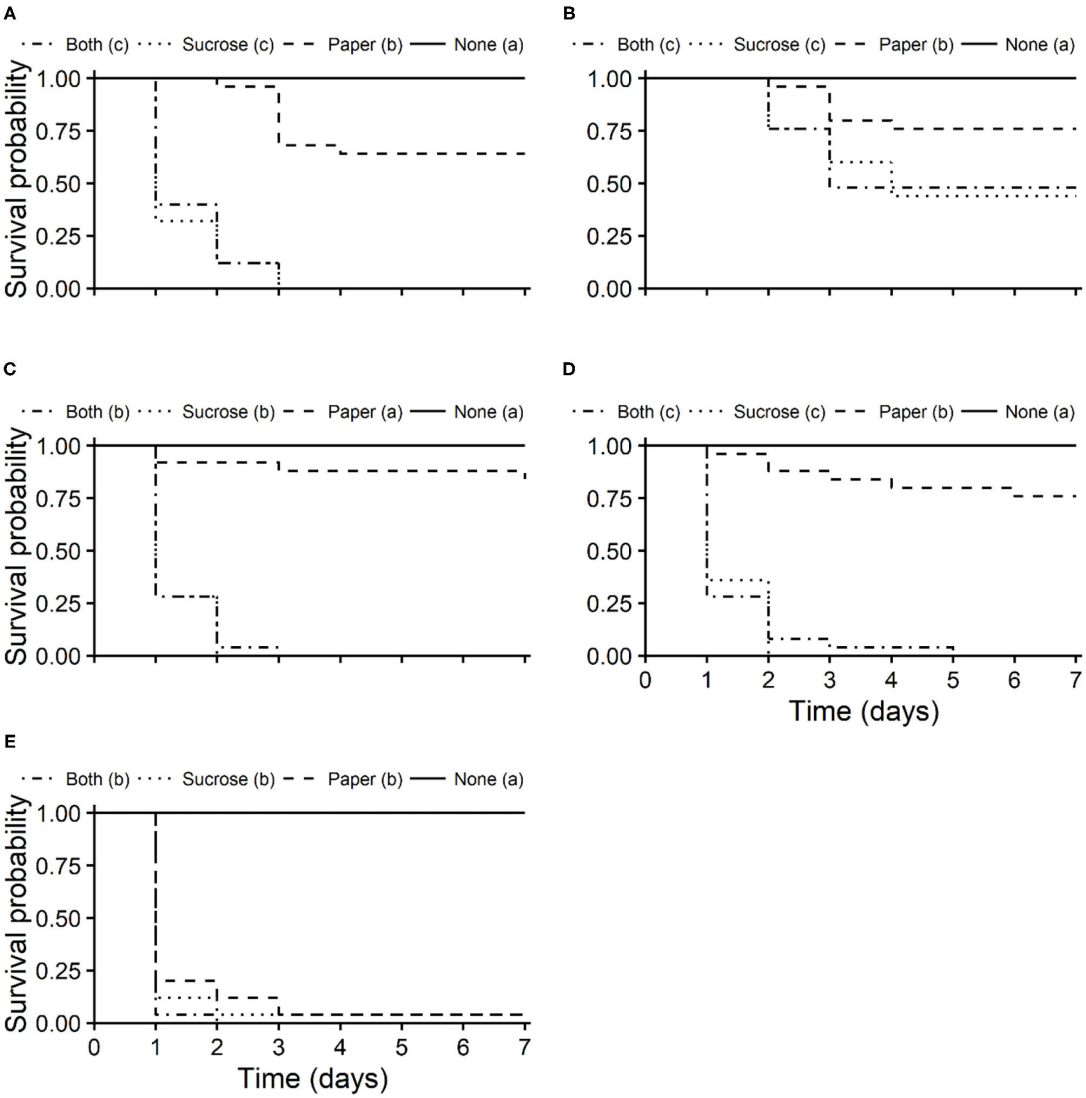
Kaplan-Meier survival probability curves of *T. japonicus* unmated females exposed to the maximum recommended field rates of pyrethrins **(A)**, azadirachtin + pyrethrins **(B)**, clothianidin **(C)**, sulfoxaflor **(D)**, and spinosad **(E)** through residual contact (paper: treated filter paper), oral (sucrose: treated sucrose solution), combined residual contact and oral exposure routes (both), and an untreated control with no insecticide (none). Different letters in parentheses in the legends indicate differences among survival curves according to the Log-Rank test adjusted with the Benjamini-Hochberg method (*P* < 0.05).

Survival curves of unmated *T. japonicus* females differed significantly among exposure routes for all insecticides (pyrethrins: χ^2^ = 102, df = 3, *P* < 0.001; azadirachtin + pyrethrins: χ^2^ = 23.1, df = 3, *P* < 0.001; clothianidin: χ^2^ = 101, df = 3, *P* < 0.001; sulfoxaflor: χ^2^ = 101, df = 3, *P* < 0.001; spinosad: χ^2^ = 91.3, df = 3, *P* < 0.001). Survival was lowest for the oral and combined oral and residual contact exposure routes, with longevities (i.e., LT_50_s) between 1 and 5 days (Figure 4). Survival of *T. japonicus* after residual contact exposure to pyrethrins, azadirachtin + pyrethrins and sulfoxaflor were higher than those exposed to the oral and combined oral and residual contact exposure routes, but less than that of the untreated control (Figures 4A,B,D, respectively). For clothianidin, the residual contact exposure route was similar to the control (Figure 4C). Survival of *T. japonicus* exposed to spinosad through all routes were similar to each other, but less than that of the control (Figure 4E).

**Figure 5:**
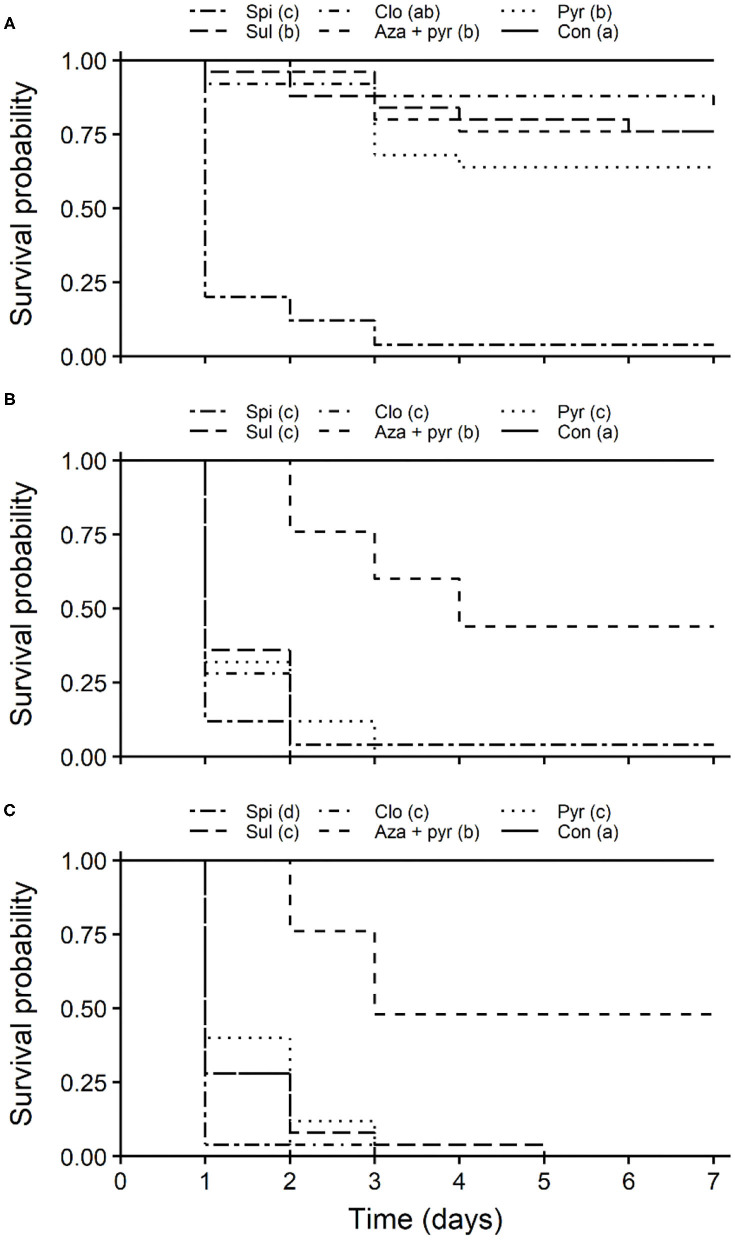
Kaplan-Meier survival probability curves of *T. japonicus* unmated females exposed to the maximum recommended field rates of five insecticides through residual contact (treated filter paper) **(A)**, oral (treated sucrose solution) **(B)**, combined residual contact and oral exposure routes **(C)**, and an untreated control with no insecticide. Different letters in parentheses in the legends indicate differences among survival curves according to the Log-Rank test adjusted with the Benjamini-Hochberg method (*P* < 0.05). Con, control; Pyr, pyrethrins; Aza + pyr, azadirachtin + pyrethrins; Clo, clothianidin; Sul, sulfoxaflor; Spi, spinosad.

Survival curves of unmated *T. japonicus* females also differed significantly among insecticides for all exposure routes (residual contact: χ^2^ = 121, df = 5, *P* < 0.001; oral: χ^2^ = 127, df = 5, *P* < 0.001; combined oral and residual contact: χ^2^ = 136, df = 5, *P* < 0.001). For the residual contact exposure route, survival was lowest for *T. japonicus* exposed to spinosad (Figure 5A). Survival of *T. japonicus* after residual contact exposure to pyrethrins, azadirachtin + pyrethrins and sulfoxaflor were greater than those exposed to the field rate of spinosad, but less than the untreated control (Figure 5A). Survival for the contact residue to clothianidin was also greater than that of spinosad, but it was similar to the control (Figure 5A). Survival of *T. japonicus* was lowest following oral exposure to clothianidin, pyrethrins, sulfoxaflor and spinosad (Figure 5B). Survival for azadirachtin + pyrethrins was higher than the other insecticides, but less than the untreated control (Figure 5B). For both oral and contact residue, survival was lowest for *T. japonicus* exposed to the field rate of spinosad (Figure 5C). Survival for pyrethrins, clothianidin and sulfoxaflor were higher than that of spinosad, but less than azadirachtin + pyrethrins. Survival of *T. japonicus* exposed to the field rate of azadirachtin + pyrethrins was greater than the other insecticides, but less than the untreated control (Figure 5C).

## Discussion

The egg parasitoid *T. japonicus* has shown promise for biological control of *H. halys*, an invasive pest of concern on multiple continents (8, 10, 11). In the present study, the lethal and sublethal effects of different rates and routes of exposure of conventional and organic insecticides to *T. japonicus* were investigated. In general, the insecticides spinosad and sulfoxaflor showed acute lethal effects, while pyrethrins, the mixture of azadirachtin and pyrethrins, and clothianidin caused sublethal effects to the wasps through residual contact exposure. However, all insecticides caused acute lethal effects when ingested by the wasps. Results of this study will inform the development of management programs of *H. halys* that are compatible with *T. japonicus*.

The highest levels of acute toxicity from residual contact exposure were observed for spinosad and sulfoxaflor. The organic insecticide spinosad was the most toxic product to *T. japonicus*. Although spinosad is considered a reduced-risk insecticide, hymenopteran parasitoids including *T. japonicus* are generally susceptible to this insecticide with moderately harmful or harmful effects (26, 28, 44, 45). Lowenstein et al. (28) found mean mortalities of *T. japonicus* higher than 90% in both laboratory and field trials, and reduced proportion of females in the population (from 0.82 to 0.05) after field application of spinosad. A similar effect was observed in the present study for all exposure routes with rapid onset of mortality (Figure 4E). *Trissolcus japonicus* mortalities above 75% and above 80% were observed within 24 h after topical exposure and ingestion, respectively, of spinosad. Thus, this organic insecticide seems to be generally harmful and therefore not compatible with *T. japonicus*.

Sulfoxaflor was also toxic to *T. japonicus* in the first experiment (i.e., residual contact toxicity) with mean mortality of 55% (Figure 1). Sulfoxaflor is generally described as an insecticide with relatively low toxicity to predators (46–48), but higher toxicity to pollinators (47) and some parasitoids (49, 50). Contrary to the first experiment (i.e., residual contact toxicity), the residual contact toxicity of sulfoxaflor (mortality <25%) was similar to that of pyrethrins, azadirachtin + pyrethrins, and clothianidin in the last experiment (i.e., different routes of exposure) (Figure 5A). Such differences in the residual contact toxicity of sulfoxaflor between these two experiments may have been due to using pooled males and females in the first experiment and only unmated females in the last experiment. Differential insecticide susceptibility between males and females has been described for parasitoids (51, 52). Although sulfoxaflor showed lower toxicity through contact exposure in the last experiment, its sublethal effects on *T. japonicus* were not investigated in this study. However, sublethal effects of sulfoxaflor to parasitoid wasps have been reported. For instance, sulfoxaflor was found to hinder the reproduction of *Eretmocerus mundus* (Mercet) (Hymenoptera: Aphelinidae) (53), *Trichogramma dendrolimi* (Matsumura), *T. ostriniae* (Pang et. Chen), and *T. confusum* (Viggiani) (Hymenoptera: Trichogrammatidae) (49). Thus, further investigations of the effects of sulfoxaflor on *T. japonicus* are recommended.

In addition to acute toxicity, sublethal effects must also be considered to more thoroughly assess the extent of potential effects of pesticides to non-target organisms (17, 20). In the present study, pyrethrins, azadirachtin + pyrethrins, and the neonicotinoid clothianidin reduced the overall longevity (as measured by overall survival curves) of *T. japonicus* at both tested rates (Figure 2), but their sublethal effects on parasitoid reproduction varied (Figure 3). Negative effects of insecticides on the longevity of parasitoids have been described in the literature for other species (25). For example, reduced longevity was also observed for females emerged from egg masses exposed to pyrethrins for *Anastatus reduvii* (Howard) (Hymenoptera: Eupelmidae) and *Telenomus podisi* (Ashmead) (Hymenoptera: Scelionidae), two egg parasitoids of stink bugs (26). In contrast, Lowenstein et al. (28) found no significant effect of pyrethrins on the longevity of *T. japonicus* after exposing the insects to insecticide-treated glass plates. The lack of an effect of pyrethrins on *T. japonicus* longevity in their study compared to the present study (Figure 2) may have been due to the lower rate of pyrethrins and/or different methods for exposure used by Lowenstein et al. (28). Interestingly, in the present study, the overall survival curve of *T. japonicus* for the higher rate of azadirachtin + pyrethrins was higher than that of the lower rate; however, the longest lived individuals between these treatments came from the lower rate (Figure 2). A similar effect was observed by Lowenstein et al. (28) for chlorantraniliprole and cyantraniliprole compared to the untreated control. Furthermore, drastically different effects were observed between the neonicotinoids thiamethoxam (i.e., longevity less than the untreated control) and imidacloprid (i.e., longevity similar to untreated control) (28). Comparisons of the results found in this study for clothianidin (Figures 2, 3) to those of Lowenstein et al. (28) with two neonicotinoids are complicated because of the different levels of toxicity of active ingredients within this group. For example, Jiang et al. (54) found variable effects of seven neonicotinoids on three species of Trichogramma parasitoid wasps (Hymenoptera: Trichogrammatidae).

Although significant differences were not observed in the proportion of female emergence resulting from exposure to the different insecticides over a 3-week period in the present study (Figure 3B), fertility was significantly reduced by clothianidin and more so at the higher rate (Figure 3C). Also, fecundity was significantly reduced by clothianidin as well as the recommended field rate of azadirachtin + pyrethrins (Figure 3D). These negative effects on *T. japonicus* reproduction may have implications at the population level of this parasitoid wasp and therefore on its biocontrol services. For instance, Stark et al. (55) suggested that mortality of ~50%, or the combination of mortality at <50% and sublethal effects on production of offspring, may be enough to cause significant impacts to natural enemy populations. Thus, the potential impacts of clothianidin and azadirachtin + pyrethrins on *T. japonicus* populations are still unknown. Taken together, these results indicate the importance of case-specific studies assessing the effects of different active ingredients and the replication of studies with the same species under different conditions. Further studies should be undertaken to assess the population-level effects of insecticides on *T. japonicus* populations, including studies conducted under field conditions.

In addition to residual contact exposure, natural enemies in the field can be exposed by ingesting contaminated prey or other food resources (e.g., honeydew, pollen, floral and extra floral nectar) (18). Oral exposure to all pesticides evaluated here caused acute toxicity to *T. japonicus*. Contrasting results for the toxicity responses of non-target organisms to different exposure routes have been documented (26, 27, 56). For instance, Haseeb and Amano (56) found that the oral toxicities to *Cotesia plutellae* (Kurdjumov) (Hymenoptera: Braconidae) of three benzoylurea insecticides showed reduced acute toxicity, although sublethal effects following ingestion of the insecticides were observed. This variability may be because of innate physiological (e.g., cuticular and digestive absorptions) and behavioral differences within species (18, 57, 58), but exposure to differing amounts of active ingredients can also be a possible explanation as suggested for *Harmonia axyridis* (Pallas) (Coleoptera: Coccinellidae) (27). In the present study, however, the amount of active ingredient ingested by each wasp is unknown so further conclusions for *T. japonicus* are not possible.

The lethal and sublethal effects of insecticides must be investigated prior to the implementation of classical or augmentative biological control because insecticides can compromise the long-term establishment of natural enemy populations in the landscape and impair the mass release of the natural enemy in agroecosystems (22). The results found in this study indicate that pyrethrins, azadirachtin + pyrethrins, clothianidin, sulfoxaflor and spinosad can impair life history parameters of *T. japonicus* through residual contact and oral routes of exposure. Therefore, insecticides should be applied with caution if *T. japonicus* is implemented for biological control of *H. halys*. In this sense, the adoption of ecological selectivity (i.e., spraying at times of low activity of the wasps or when they are not present, and reducing the contamination of food sources) is recommended to increase the compatibility between biological and chemical control (18, 59). The present study advances the knowledge for conservation of *T. japonicus* to ensure its efficacy as a biological control agent for *H. halys* under conventional and organic farming systems.

## Data Availability Statement

The raw data supporting the conclusions of this article will be made available by the authors, without undue reservation.

## Author Contributions

WH and RK: conceptualization, resources, project administration, and funding acquisition. RK, WH, and SH: methodology. AR and SH: investigation and formal analysis. AR and RK: writing original draft preparation. AR, RK, and WH: writing. RK: supervision. All authors have read and agreed to the published version of the manuscript.

## Conflict of Interest

The authors declare that the research was conducted in the absence of any commercial or financial relationships that could be construed as a potential conflict of interest.
